# Connective Tissue Disease-Associated Pulmonary Arterial Hypertension in Southern Taiwan: A Single-Center 10-Year Longitudinal Observation Cohort

**DOI:** 10.3390/healthcare9050615

**Published:** 2021-05-20

**Authors:** Chun-Hsin Wu, Chun-Yu Lin, Chih-Hsin Hsu, Sheng-Hsiang Lin, Chia-Tse Weng

**Affiliations:** 1 Institute of Clinical Medicine, College of Medicine, National Cheng Kung University, Tainan 701, Taiwan; shlin922@mail.ncku.edu.tw; 2 Division of Rheumatology, Department of Internal Medicine, National Cheng Kung University Hospital, College of Medicine, National Cheng Kung University, Tainan 701, Taiwan; weng.ct@gmail.com; 3 Department of Internal Medicine, National Cheng Kung University Hospital, College of Medicine, National Cheng Kung University, Tainan 701, Taiwan; linchunyumed@gmail.com; 4 Division of Cardiology, Department of Internal Medicine, National Cheng Kung University Hospital, College of Medicine, National Cheng Kung University, Tainan 701, Taiwan; chihhsinhsu@gmail.com

**Keywords:** pulmonary arterial hypertension, connective tissue disease, mean pulmonary arterial pressure

## Abstract

*Background*: Pulmonary arterial hypertension (PAH) is a life-threatening disease with different etiologies and outcomes. We aimed to explore differences in clinical features and outcomes of idiopathic PAH (iPAH) and connective tissue disease-related PAH (CTD-PAH) in Taiwanese patients and determine risk factors for mortality. *Methods*: We retrospectively reviewed the medical records of patients with right-sided heart catheterization-diagnosed PAH between January 2005 and December 2015. The iPAH (*n* = 31) and CTD-PAH (*n* = 14) patients were enrolled and followed until December 31, 2019. Between-group comparisons were conducted. Potential predictors of the mortality of PAH were determined using the Cox proportional hazard regression model. *Results*: CTD-PAH patients had higher levels of N-terminal pro-brain natriuretic peptide (NT-proBNP) and lower predicted diffusing capacity of carbon monoxide (DLCO) than iPAH patients. The mortality rates were similar between CTD-PAH and iPAH (21.4% vs. 22.6%, *p* = 0.99). A mean pulmonary arterial pressure (mPAP) > 46 mmHg was a predictor of PAH-induced mortality (adjusted hazard ratio 21.8, 95% confidence interval 2.32–204.8). *Conclusions*: A higher mPAP level, but not underlying CTDs, imposed a significantly increased risk of mortality to patients with PAH.

## 1. Introduction

Pulmonary arterial hypertension (PAH) is a life-threatening disease defined by a mean pulmonary arterial pressure (mPAP) ≥ 25 mmHg at rest using right-sided heart catheterization (RHC) [1], end-expiratory pulmonary artery wedge pressure (PAWP) ≤ 15 mmHg, and pulmonary vascular resistance (PVR) > 3 Wood units. PAH is categorized as group 1 pulmonary hypertension (PH) and consists of idiopathic PAH (iPAH, group 1.1); heritable PAH (groups 1.2.1-3); PAH due to drugs or toxins (group 1.3); and PAH associated with connective tissue disease (CTD, group 1.4.1), HIV infection (group 1.4.2), portal hypertension (group 1.4.3), congenital heart diseases (group 1.4.4), and schistosomiasis (group 1.4.5) [2]. A comprehensive patient evaluation is essential for a definitive diagnosis.

The Registry to Evaluate Early and Long-Term PAH Disease Management (REVEAL), the largest US cohort of patients with RHC-diagnosed PAH conducted between 2006 and 2007, provides comprehensive epidemiologic information for group 1 PAH and disease characteristics for each subgroup [3]. According to the REVEAL registry, patients with iPAH comprised 46.3% of the entire population, while CTD-PAH accounted for 25.3% of patients. Patients with CTD-PAH had higher B-type natriuretic peptide (BNP) levels and lower diffusing capacity of carbon monoxide (DLCO) but lower mPAP levels than those of iPAH patients. One-year survival and freedom from hospitalization were lower in patients with CTD-PAH than in those with iPAH. However, these findings cannot be extrapolated to Asian patients, since autoimmune rheumatic diseases are ethnically and epidemiologically diverse worldwide. The distribution of CTD in the setting of PAH differs between Asian and Western countries. Of the 641 patients with CTD-PAH in the REVEAL registry, 62.2% were diagnosed with systemic sclerosis (SSc), 17.2% with systemic lupus erythematosus (SLE), and 8.1% with as-mixed connective tissue disease (MCTD). However, SLE was more prevalent than SSc among patients with CTD-PAH in China, Korea, and Japan [4,5,6]. Therefore, the characteristics and prognosis of patients with CTD-PAH may differ between Asian and Western countries.

Hemodynamic parameters, such as pulmonary arterial pulse pressure [7] or the pulmonary–systemic pulse pressure ratio [8], had a prognostic impact on patient outcomes; nonetheless, none of these parameters have been validated in Taiwanese patients. Therefore, we aimed to compare the clinical features and survival of patients with CTD-PAH and iPAH from a tertiary referral center and determine potential risk factors for PAH mortality. We also sought to compare clinical characteristics of SLE and non-SLE-related CTD-PAH, as CTD-PAH in the majority of patients was due to SLE in the Asian population. We expected that these results would provide valuable information concerning the monitoring of SLE-PAH in Asian countries.

## 2. Methods

### 2.1. Data Source and Study Subjects

A comprehensive retrospective medical record review was performed for patients diagnosed with PAH at the National Cheng Kung University Hospital (NCKUH) from January 2005 to December 2015. This study complied with the Declaration of Helsinki. The Institutional Review Board of NCKUH approved the study protocol (A-ER-104-344), which waived the requirement for written informed consent for data analysis due to the study’s retrospective nature.

Patients (aged ≥ 16 years) were identified as having PAH only if they had undergone RHC to measure hemodynamic parameters and had fulfilled the definition of PAH as mPAP ≥ 25 mmHg, PAWP ≤ 15 mmHg, and PVR > 3 Wood units. Underlying CTDs among patients with PAH were ascertained by classification criteria, including the 1982 American College of Rheumatology classification for systemic lupus erythematosus (SLE), the 2013 American College of Rheumatology–European League Against Rheumatism criteria for systemic sclerosis for SSc, and the Alarcón-Segovia criteria for mixed connective tissue disease (MCTD). Patients with PAH without other associated diseases or conditions were grouped as iPAH, while patients diagnosed with PH beyond group 1 were excluded. The index date was the date of the first diagnosis of PAH. Patients with CTD-PAH and iPAH with an index date between January 2005 and December 2015 were enrolled and followed from the index date until death or 31 December 2019.

### 2.2. Covariates and Comorbidities

Functional and hemodynamic parameters, including serum level of N-terminal pro-brain natriuretic peptide (NT-proBNP), estimated pulmonary arterial systolic pressure (ePASP) measured by transthoracic echocardiography (TTE), mPAP measured by RHC, and DLCO level measured by standardized pulmonary function protocol were recorded.

The selected comorbidities included chronic kidney disease (CKD, defined as estimated glomerular filtration rate < 60 mL/min/1.73 m^2^), hypertension, diabetes mellitus, and dyslipidemia. These comorbid conditions were identified based on laboratory results or history of prescription medication before the date of PAH diagnosis.

### 2.3. Autoantibody Detection

Antinuclear antibody (ANA) was detected by indirect immunofluorescence assay techniques using human epithelial tumor cell lines (Hep-2), with a titer of ≥1:80 by Hep-2 immunofluorescence being defined as positive. Extractable nuclear antigen (ENA) was tested by the ELISA method using UniCAP-100 (Phadia, Thermo Fisher Scientific Inc., Uppsala, Sweden), which included assays detecting antibodies directed at double-strand DNA, Sm antigens, ribonucleoprotein (RNP), Sjögren’s syndrome type A (Ro), and Sjögren’s syndrome type B (La) antigens.

### 2.4. Treatment

We recorded the PAH-specific therapy, including prostacyclin agonists (epoprostenol, treprostinil, iloprost), endothelin receptor antagonists (bosentan, ambrisentan), and phosphodiesterase type 5 (PDE5) inhibitors (sildenafil). Users of PAH-specific therapy were defined by dispensed prescription for 3 months or more during the study period.

### 2.5. Statistical Analyses

Continuous measurements with a normal distribution are expressed as the mean and standard deviation; measurements from non-Gaussian distribution are presented as the median with 25–75% interquartile range. Categorical variables are expressed as a number and percentage. The independent Student’s t test, Mann–Whitney test, and chi-square test were used for between-group comparisons as appropriate. Survival rates were calculated using the Kaplan–Meier method, and the log-rank test was used to determine differences between survival curves. Cox proportional hazard regression was used to identify predictors of mortality; these results were reported as hazard ratios (HRs) with 95% confidence intervals (CI). A two-sided *p*-value < 0.05 was considered statistically significant. All statistical analyses were performed using SPSS software version 17.0 (SPSS Inc., Chicago, IL, USA).

## 3. Results

### 3.1. Demographic Features of Patient Characteristics

**Figure 1:**
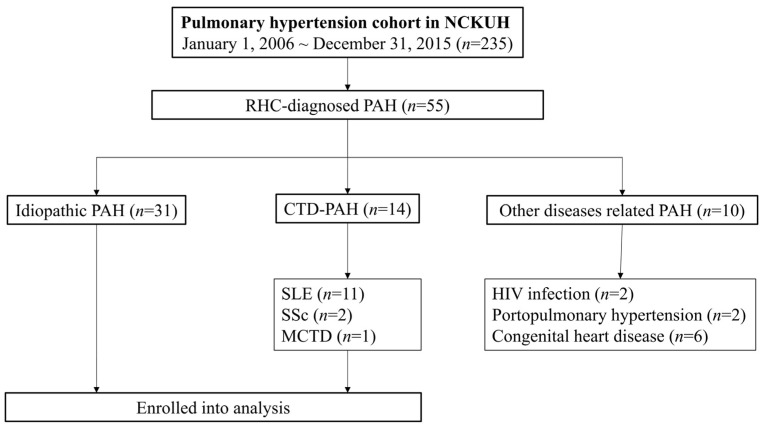
Study flowchart.

Fifty-five patients with RHC-confirmed PAH were enrolled during the study period: 31 were classified as iPAH. Among the 14 patients with CTD-PAH, 11 (78.6%) had SLE, 2 (14.3%) had SSc, and 1 (7.1%) patient had MCTD. Non-CTD-related PAH included two cases associated with HIV infection, two with portopulmonary hypertension, while six cases presented congenital heart disease-related CTD (Figure 1). A total of 10 patients with PAH died, including seven patients with iPAH and three patients with SLE-PAH.

Demographic and clinical information for the patients with CTD-PAH and iPAH are summarized in Table 1. Female patients accounted for approximately half of the iPAH-group, whereas patients with CTD-PAH were predominately female. Compared with iPAH, patients with CTD-PAH were younger at the onset of PAH. As expected, the prevalence rates of positive ANA and ENA were significantly higher in the CTD-PAH group. Only two patients with iPAH had a titer of ANA of 1:80, but none had detectable ENA. The presence of comorbidities was not significantly different between the two groups. There was no statistically significant difference in PAH-related mortality between the two groups (21.4% vs. 22.6%, *p* = 0.99).

Compared with iPAH, patients with CTD-PAH had significant higher levels of NT-proBNP and lower DLCO. As for hemodynamic parameters, the estimated pulmonary arterial systolic pressure (ePASP) levels, measured via transthoracic echocardiography and the mean PAP and determined by RHC, were not significantly different between the two groups.

PDE5 inhibitors were the most commonly used drugs among all groups, accounting for 64.5% and 100% of patients with iPAH and CTD-PAH, respectively.

### 3.2. Characterizations of Patients with SLE and PAH

The clinical features of the SLE-PAH group are listed in Table 2. Here, 10 out of 11 patients developed PAH following the diagnosis of SLE, while only one patient developed SLE and PAH in the same year (within an interval of 4 months). The median duration from SLE diagnosis to PAH diagnosis was 11 years (interquartile range, 4–16). Among 11 patients, five had lupus nephritis, while eight patients (72.7%) had anti-RNP autoantibodies. Three patients experienced PAH-related mortality during the study period, all of whom died within 4 years of the PAH diagnosis.

### 3.3. Survival Analysis of Patients with PAH

The 1-, 3-, and 5-year survival rates of the patients with PAH overall were 93.3%, 82.2%, and 80.0%, respectively (Supplementary Figure S1). Baseline characteristics were not significantly different between the surviving and deceased patients. With respect to hemodynamic parameters, deceased patients with PAH tended to have higher levels of mPAP than patients with long-term survival (51.9 ± 7.8 vs. 43.0 ± 14.9, *p* = 0.069) (Table 3). There was no significant difference in the rates of exposure to PAH-specific therapies among the survivors and the dead.

**Figure 2:**
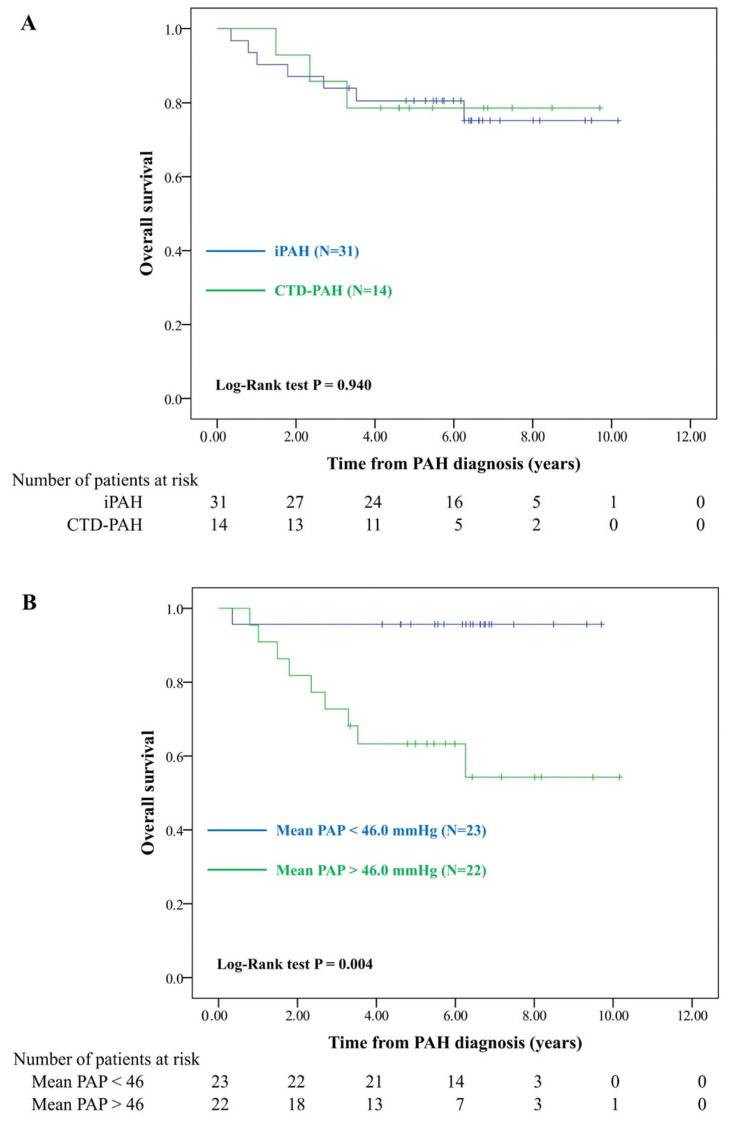
Overall survival of pulmonary arterial hypertension in different subgroups. (**A**) Patients with iPAH vs. CTD-PAH. (**B**) Patients with mPAP < 46 mmHg vs. >46 mmHg; iPAH: idiopathic pulmonary arterial hypertension; CTD-PAH: connective tissue disease-related PAH; mPAP: mean pulmonary arterial hypertension.

Patients with CTD and SLE did not exhibit a higher PAH-induced mortality rate (Figure 2A and Supplementary Figure S2) than patients with iPAH. Kaplan–Meier curve analysis showed that patients with mPAP greater than the median levels (46.0 mmHg) had significantly lower long-term survival (Figure 2B). The multivariable Cox model showed that high mPAP was a significant risk factor for mortality (adjusted HR 21.81, 95% CI 2.32–204.88) (Table 4).

## 4. Discussion

To the best of our knowledge, this is the first study directly comparing RHC-diagnosed PAH among different subgroups from a single medical center. We found that patients with CTD-PAH were younger at disease onset and had higher NT-proBNP levels and lower DLCO than patients with iPAH. Regarding measurable hemodynamic parameters, patients in both groups had similar ePASP and mPAP levels. Outcomes were not significantly different between patients with CTD-PAH and iPAH, and a high mPAP was a risk factor for PAH-related mortality.

PAH is a rare disease with an estimated incidence of 2.0–7.6 cases per million and a prevalence ranging from 10.6 to 26 per million adults based on several cohort studies from Europe and North America [9,10]. In previous cohort studies, iPAH comprised 30–50% of patients with PAH, whereas CTD-PAH was the second most prevalent cause at 15–30% [11]. A recent epidemiologic report assessing the Taiwanese National Health Insurance Research Database (NHIRD) showed that the population with idiopathic PH and CTD-PH (17.31% vs. 16.76%) was very similar [12]. This report might have overestimated the prevalence of PAH for the following reasons. First, the data were based on the International Classification of Diseases, Ninth Revision, Clinical Modification (ICD-9-CM) codes (416.0 primary PH and 416.8 other chronic pulmonary heart diseases), without validation of RHC implementation. Second, patients with CTD, especially SLE, could have had inflammation of the myocardium, pulmonary parenchyma, or complications by pulmonary thromboembolism, resulting in group 2, 3, or 4 PH, respectively. If those pathologic conditions were not excluded by a thorough investigation, overestimation of PAH might exist among CTD-PAH. Among the 55 cases identified in our study, 31 patients were diagnosed with iPAH (56.4%) and 14 with CTD-PAH (25.6%). We confirmed that CTD-PAH is the second most prevalent among disease-associated PAH in the Asian population.

In our CTD-PAH group, the majority had SLE (11/14 patients, 78.6%); this is consistent with other Asian registries [4,5,6] and differs from the Western cohort, in which SSc comprises most of the population [9]. The difference may result from the different prevalences of connective tissue disease between Western countries and Asia [11,13,14]. The prevalence of PAH in CTD varies among disease entities. The most studied was SSc, which ranged from 7.85% to 19% among patients, as confirmed by RHC [15,16,17]. During this study period, only two RHC-diagnosed patients with PAH among 56 SSc patients were identified. The estimated prevalence was 3.57%, with an additional three patients with PH diagnosed by transthoracic echocardiography (TTE). The actual prevalence of PAH in patients with SLE is unknown; the published data are highly variable owing to differences in diagnostic methods used and the nature of studied cohorts. Chen et al. reported 19 CTD-PAH cases in a 2-year Taiwanese cohort using a cut-off of the right ventricular systolic pressure (RVSP) ≥ 40 mmHg [18]. Li et al. reported that the prevalence in Chinese patients with SLE was approximately 3.8% using a cut-off of systolic pulmonary artery pressure (sPAP) ≥ 40 mmHg [19]. Another study estimated that 2.13% of patients with SLE developed PAH [20]. However, none of the patient diagnoses in the above studied population were validated by RHC measurement; thus, PH might be misclassified as PAH. If RHC had been required for diagnosis, the prevalence would have been lower. Ruiz-Irastorza et al. adopted a diagnostic strategy in patients with SLE with possible PH defined as two consecutive sPAP values of ≥40 mmHg by TTE; none of the patients had PAH eventually confirmed by RHC [21]. In a 2-year cohort study including 152 SLE patients [22], only three PAH and one possible early PAH, defined as exercise-induced pulmonary artery pressure increase with PAWP < 20 mmHg, were found. There were 1074 patients with SLE in our study, and only 11 RHC-diagnosed patients with PAH were identified; thus, the estimated prevalence was quite low (1.02%). Epidemiological data relative to MCTD is far more limited; in a 3-year Norwegian nationwide cohort, two PAH cases were identified among 147 adult patients with MCTD [23]. In our cohort, one of the 11 patients with MCTD had RHC-diagnosed PAH, while another had elevated sPAP measured by TTE. Overestimation of PAH may lead to unnecessary medical treatment or inappropriate management if patients have other causes of PH, such as interstitial lung diseases, left heart diseases, or pulmonary thromboembolism. Instead, a multidisciplinary approach to susceptible patients should be conducted to optimize patient benefits. Among patients with SLE-PAH, we found a high prevalence of anti-RNP antibody, which is an independent risk factor for PAH development in patients with SLE [24]. We suggest that a level of ePASP > 45 mmHg associated with anti-RNP positivity should prompt regular monitoring of PAH development.

PAH treatment is thought to be less effective in patients with CTD, despite appropriate vasoactive therapy with combination immunosuppressants [25]. This might be attributable to the variant subgroup of patients with CTD in Western cohorts discussed above. Subjects enrolled for treatment efficacy are mostly those with SSc-PAH. SSc is characterized by fibrosis of the internal organs and vasculopathy. Fibrosis is a late process of inflammation and cell proliferation that eventually results in vascular remodeling and vasoconstriction. This pathophysiology may lead to immunosuppressive therapy inefficacy. Pulmonary fibrosis, another hallmark manifestation of SSc, is also less responsive to novel antifibrotic therapy [26,27]. Therefore, it is reasonable to speculate that patients with SSc-PAH exhibited a poor response to standard vasoactive therapy. In contrast, SLE is a systemic disease involving acute and chronic inflammation of multiple organs, including the arterial or venous vasculature. Pulmonary vasculitis leads to endothelial injury, vascular damage, and subsequent elevated pulmonary arterial pressure (PAP) [28]. Moreover, patients with SLE usually experience acute flares with organ inflammation that physicians tend to expose to intensive immunosuppressive therapy, which is not recommended for most patients with SSc, which may help to ameliorate inflammation of the pulmonary vasculature. This different strategy of SLE management could have additional benefits to vasoactive therapy and may contribute to improved outcomes for Asian patients with CTD-PAH.

The REVEAL registry reported worse outcomes in patients with CTD-PAH than in those with iPAH [29]. The 1-, 3-, and 5-year survival rates of iPAH were 88.4%, 73.7%, and 64.3%, respectively, compared with survival rates of 79.5%, 57.1%, and 43.7%, respectively, among patients with CTD-PAH. Among the CTD-PAH subgroups, one-year survival rates were worse in patients with SSc-PAH than in patients with SLE-PAH (82% vs. 94%) [30]. However, the survival of patients with PAH overall has substantially improved over the past decade. In a recently published cohort study from Singapore, the 1-, 3-, and 5-year survival rates of iPAH were 96.0%, 86.4%, and 79.0%, respectively [31]. Another Japanese study of 141 idiopathic or heritable patients with PAH described 3-, 5-, and 10-year survival rates of 92.1%, 85.8%, and 69.5%, respectively [32]. The outcomes of SLE-PAH have also improved. In a recent single hospital cohort study from Korea, the 3- and 5-year survival rates after PAH diagnosis were 88.8% and 86.1%, respectively [33]. A Chinese multicentric cohort study reported 3- and 5-year survival rates of 84.8% and 72.9%, respectively [34]. Among our 11 patients with SLE-PAH, three died from PAH-related causes within 5 years after PAH onset. The 5-year survival rate for patients with SSc-PAH in the REVEAL registry was 40% compared with 61.7% from a recent Australian cohort [35] and 63% from another recent North American registry [36]. The literature for Asian patients with SSc-PAH is limited because of its low prevalence. Our two patients with SSc-PAH survived for more than 5 years until the end of the study. The advances of PAH treatment and adherence to management guidelines have contributed to considerable improvements in patient care.

Elevated PAP causes right ventricular dysfunction, compromises biventricular integrity, and has negative hemodynamic effects on the pulmonary and systemic circulation leading to adverse outcomes. A classification and regression trees model illustrated that the increase in mPAP was proportional to poor PAH outcome [37]. Our analysis revealed that patients with mPAP > 46 mmHg had a significantly worse prognosis, which is comparable with the findings of a Japanese study [32].

NT-proBNP is a marker of right ventricular dysfunction secreted by cardiomyocytes following ventricular overload. The serum NT-proBNP level is an integral variable of risk stratification in PAH and is included in the scoring system developed based on the REVEAL registry. A post hoc analysis from the GRIPHON study, a double-blind randomized placebo-controlled phase III study that assessed the safety and efficacy of selexipag in patients with PAH, established the prognostic relevance of NT-proBNP levels and their association with treatment response [38]. We found no significant prognostic impact on survival in the present study, likely owing to the small sample size and missing data.

Recent data from the prospective Pulmonary Hypertension Assessment and Recognition of Outcomes in the Scleroderma (PHAROS) registry demonstrated that a low DLCO was a predictor of mortality for patients with SSc-PAH [39]. However, this was not observed in other PAH subgroups from previous cohort studies. In the between-group comparison, a significantly lower DLCO was not associated with poor outcomes in patients with CTD-PAH. DLCO on survival requires further research.

The strength of our study is that the enrolled study subjects all had RHC-diagnosed PAH. Each patient underwent a thorough investigation to avoid misclassification in other groups of PH. We provided direct comparisons between CTD-PAH and iPAH. All patients in our cohort were managed by the same multidisciplinary team, minimizing inter- or intraobserver biases.

Our study had some limitations. First, the small sample size could limit the significance of several potential prognostic predictors, such as DLCO, NT-proBNP, and CKD. Second, due to the retrospective design, some patients had missing data, including serologic parameters or pulmonary function test results. The follow-up assessments were not standardized either. Third, due to the small number of patients in each group of CTD-PAH, a subgroup analysis could not be conducted.

## 5. Conclusions

Our single-hospital PAH cohort showed a commensurate survival with that of modern Western and Asian registries. Although NT-proBNP levels were higher and DLCO was lower in patients with CTD-PAH than in patients with iPAH, these differences did not negatively influence the survival outcome of patients with CTD-PAH. Our results indicate that high baseline mPAP was a poor prognostic factor for mortality in patients with PAH. A comprehensive risk assessment with multidisciplinary management should be conducted to avoid devastating outcomes.

## Figures and Tables

**Table 1:** Baseline characteristics of the patients with PAH.

Characteristics	CTD-PAH		iPAH	*p* Value: CTD-PAH vs. iPAH
	All (*N* = 14)	SLE (*N* = 11)	(*N* = 31)	
Female	13 (92.9)	11 (100)	17 (54.8)	0.016
Age at PAH diagnosis, years	38.6 (10.6)	38.7 (9.7)	47.7 (14.4)	0.040
NT-proBNP ^a^ (pg/mL) (25–75% IQR)	1089.3 (636.4–3644.0)	1264.0 (684.1–3638.0)	441.5 (213.1–2125.0)	0.039
ePASP ^b^ (mmHg)	67.0 (22.5)	71.1 (23.3)	81.0 (31.2)	0.141
mPAP (mmHg)	42.8 (9.5)	43.2 (9.5)	46.4 (15.2)	0.322
DLCO ^c^ (% of predicted)	55.3 (11.8)	53.5 (11.6)	75.5 (25.4)	0.014
ANA positive	13 (92.9)	11 (100)	2 (6.7)	<0.001
ENA positive	13 (92.9)	11 (100)	1 (3.3)	<0.001
PAH-induced mortality	3 (21.4)	3 (27.3)	7 (22.6)	0.99
Mean follow-up years (min–max)	5.3 (1.5–9.7)	4.8 (1.5–9.7)	5.6 (0.4–10.2)	0.715
Comorbidity				
CKD	2 (14.3)	2 (18.2)	9 (29.0)	0.458
Hypertension	4 (28.6)	4 (36.4)	7 (22.6)	0.717
Diabetes mellitus	0 (0.0)	0 (0.0)	5 (16.1)	0.305
Dyslipidemia	2 (14.3)	2 (18.2)	4 (12.9)	1.000
PAH-specific therapy				
Prostacyclin agonists	1 (7.1)	0 (0)	6 (19.4)	0.407
ERA	1 (7.1)	1 (9.1)	10 (32.3)	0.132
PDE5 inhibitor	14 (100)	11 (100)	20 (64.5)	0.010

Data are presented as means (S.Ds) or n (%) unless otherwise noted. CTD: connective tissue disease; SLE: systemic lupus erythematosus; PAH: pulmonary arterial hypertension; iPAH: idiopathic pulmonary arterial hypertension; NT-proBNP: amino-terminal pro-B-type natriuretic peptide; ePASP: estimated pulmonary arterial systolic pressure; mPAP: mean pulmonary arterial hypertension; DLCO: diffusing capacity of the lungs for carbon monoxide; ANA: antinuclear antibody; ENA: extractable nuclear. antigen. CKD: chronic kidney disease. ERA: endothelin receptor antagonists; PDE5: phosphodiesterase type 5; ^a^ 27 in the iPAH; ^b^ 30 in the iPAH; ^c^ 12 cases in the CTD-PAH and 23 the in iPAH.

**Table 2:** Selected clinical features of the SLE-PAH group.

No.	Sex	Age at SLE	Age at PAH	Time to PAH Onset (year)	LN	Anti-RNP	APL			NT-proBNP (pg/mL)	mPAP (mmHg)	DLCO (%)	Outcome
							LAC	aCL	β2GP1				
1	F	16	17	1	-	+	-	-	-	3638	58	51	Died at age 19 years
2	F	41	46	5	+	-	-	-	-	684.1	36	54	Survival
3	F	24	42	18	-	-	-	-	-	2029	45	63	Survival
4	F	30	34	4	+	+	-	-	-	109.8	47	65	Died at age 35 years
5	F	43	53	10	-	-	-	-	-	3424	52	38	Died at age 56 years
6	F	29	29	0.3	+	+	-	-	-	422.1	55	67	Survival
7	F	19	37	18	-	+	+	-	-	1264	32	41	Survival
8	F	26	38	12	+	+	+			8346	38	N/A	Survival
9	F	30	46	16	-	+	-	-	-	914.5	44	63	Survival
10	F	33	44	11	-	+	-	-	-	887.9	40	57	Survival
11	F	29	40	11	+	+	-	-	-	7048	28	36	Survival

F: female; SLE: systemic lupus erythematosus; LN: lupus nephritis; Anti-RNP: anti-ribonucleoprotein antibody; APL: antiphospholipid; LAC: lupus anticoagulant; aCL: anticardiolipin antibody. Β2GP1: anti-β2glycoprotein 1; NT-proBNP: amino-terminal pro-B-type natriuretic peptide; mPAP: mean pulmonary arterial hypertension; DLCO: diffusing capacity of the lungs for carbon monoxide; N/A: not applicable.

**Table 3:** Baseline demographic information and characteristics of PAH patients with mortality and survival.

	Mortality (*N* = 10)	Survival (*N* = 35)	*p* Value
Female	7 (70)	23 (65.7)	1.000
Age at PAH diagnosis	43.2 (16.6)	45.3 (13.2)	0.676
CTD	3 (30)	8 (22.9)	0.687
NT-proBNP ^a^ (pg/mL) (25–75% IQR)	1867.5 (535.3–3147.5)	546.5 (257.9–2077.0)	0.235
ePASP ^b^ (mmHg)	81.9 (16.6)	74.9 (32.0)	0.516
mPAP (mmHg)	51.9 (7.8)	43.0 (14.9)	0.069
DLCO ^c^ (% of predicted)	55.9 (22.1)	71.7 (23.2)	0.113
Comorbidity			
CKD	4 (40.0)	7 (20.0)	0.228
Hypertension	3 (30)	8 (22.9)	0.687
Diabetes mellitus	1 (10)	4 (11.4)	1.000
Dyslipidemia	1 (10)	5 (14.3)	1.000
PAH-specific therapy			
Prostacyclin agonists	3 (30)	4 (11.4)	0.172
ERA	2 (20)	9 (25.7)	1.000
PDE5 inhibitor	8 (80)	26 (74.3)	1.000

Data are presented as means (S.Ds) or n (%) unless otherwise noted. CTD: connective tissue disease; PAH: pulmonary arterial hypertension; iPAH: idiopathic pulmonary arterial hypertension; NT-proBNP: amino-terminal pro-B-type natriuretic peptide; ePASP: estimated pulmonary arterial systolic pressure; mPAP: mean pulmonary arterial hypertension; DLCO: diffusing capacity of the lungs for carbon monoxide; ANA: antinuclear antibody; ENA: extractable nuclear. antigen. CKD: chronic kidney disease. ERA: endothelin receptor antagonists; PDE5: phosphodiesterase type 5; ^a^ 8 cases of mortality and 33 of survival; ^b^ 10 cases of mortality and 34 of survival; ^c^ 7 cases of mortality and 28 of survival.

**Table 4:** Crude and adjusted hazard ratios of predictors of mortality among patients with pulmonary arterial hypertension.

	Crude HR (95% CI)	*p* Value	Adjusted HR (95% CI) ^a^	*p* Value
CTD	0.95 (0.24–3.68)	0.940	3.29 (0.66–16.35)	0.144
Mean PAP > 46.0 mmHg	11.36 (1.43–89.98)	0.021	21.81 (2.32–204.88)	0.007

^a^ Adjusted for age and sex. HR: hazard ratio; CTD: connective tissue disease; PAP: pulmonary arterial hypertension.

## Data Availability

The data presented in this study are available on request from corresponding author.

## Supplementary Materials

The following are available online at https://www.mdpi.com/article/10.3390/healthcare9050615/s1: Figure S1: Overall survival of all patients with pulmonary arterial hypertension. Figure S2: Overall survival of pulmonary arterial hypertension in patients with iPAH vs. SLE-PAH.
