# Supplementary material for: Connective Tissue Disease-Associated Pulmonary Arterial Hypertension in Southern Taiwan: A Single-Center 10-Year Longitudinal Observation Cohort
Source: Healthcare (Basel). 2021 May 20;9(5):615. doi: 10.3390/healthcare9050615 (PMC8160731; doi:10.3390/healthcare9050615)
Supplement: Supplementary file 1 [file healthcare-09-00615-s001.zip › Figure S2.pdf]

Figure S2

Overall survival of pulmonary arterial hypertension in patients with iPAH vs SLE-PAH.

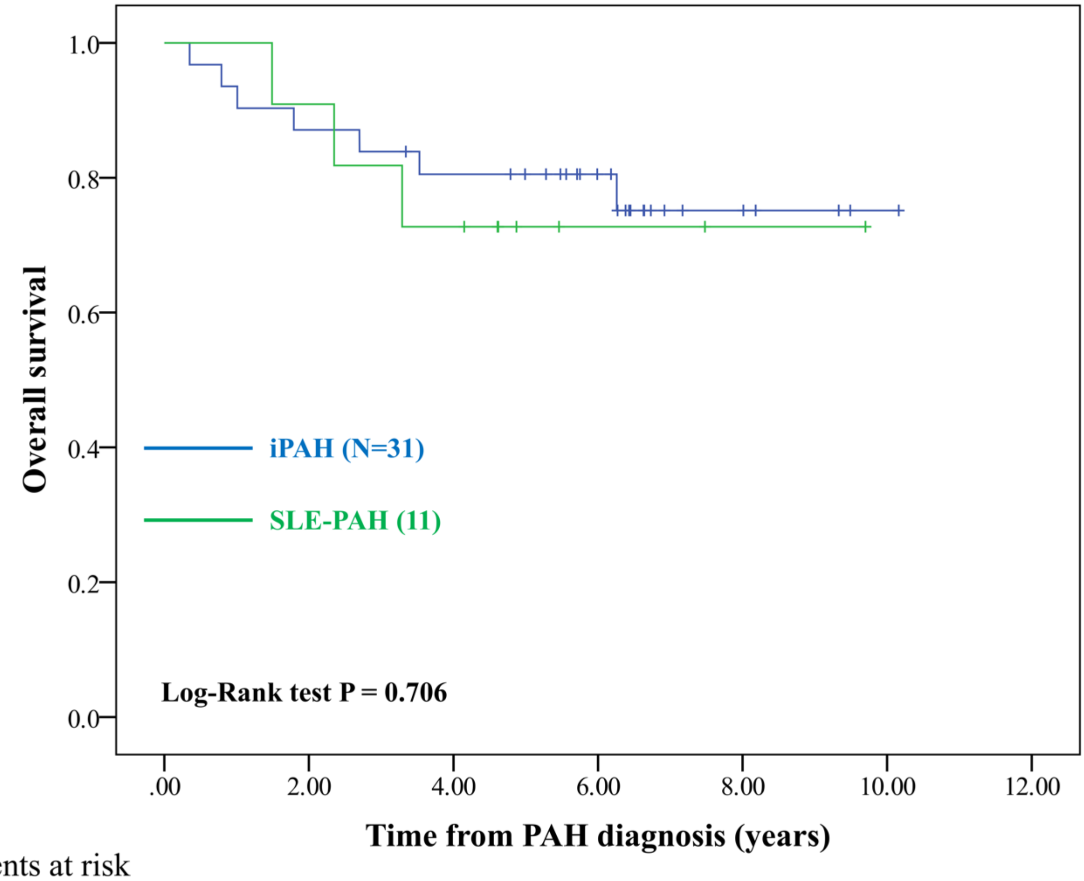

Number of patients at risk

iPAH	31	27	24	16	5	1	0
SLE-PAH	11	10	8	2	2	0	0
