# Supplementary figures and images for: Connective Tissue Disease-Associated Pulmonary Arterial Hypertension in Southern Taiwan: A Single-Center 10-Year Longitudinal Observation Cohort
Source: Healthcare (Basel). 2021 May 20;9(5):615. doi: 10.3390/healthcare9050615 (PMC8160731; doi:10.3390/healthcare9050615)

**Figure S1**

Overall survival of all patients with pulmonary arterial hypertension

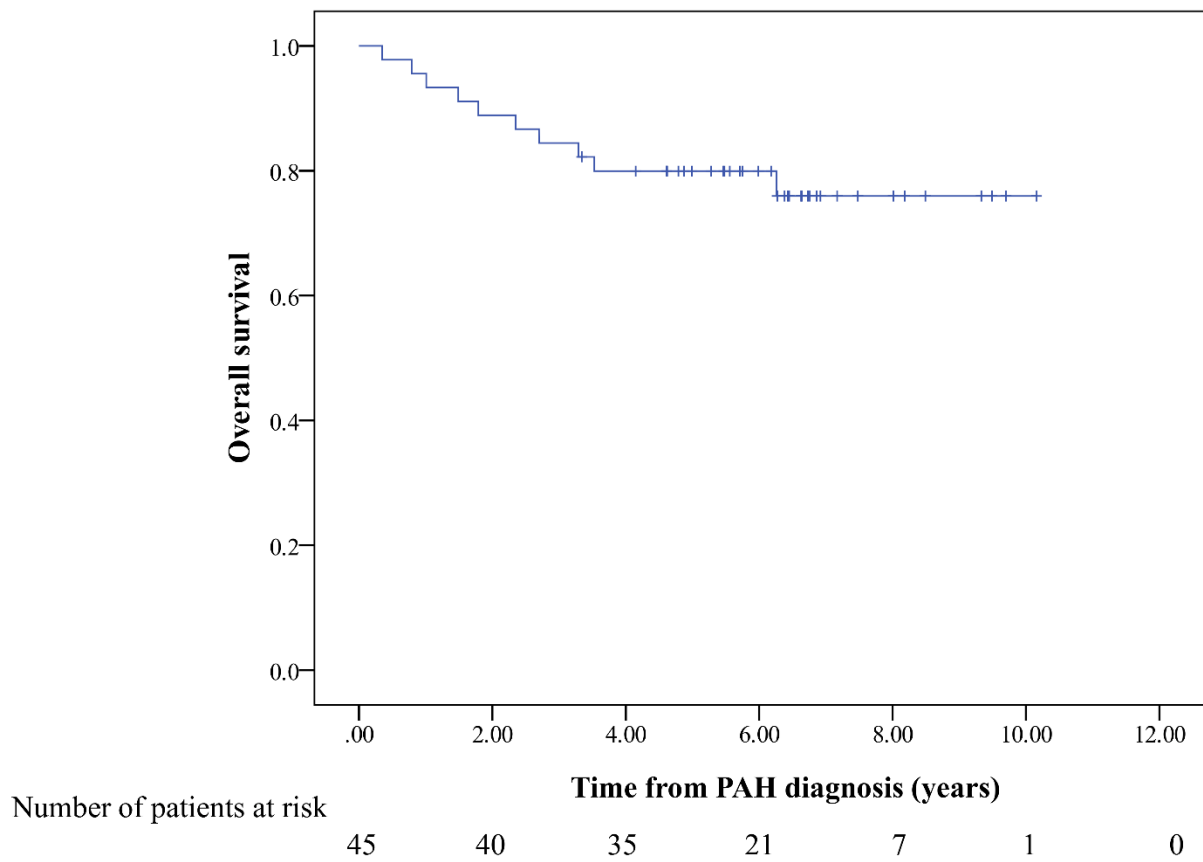

Supplement: Supplementary file 1 [file healthcare-09-00615-s001.zip › Figure S1.pdf]
